# Complex regulation of ADAR-mediated RNA-editing across tissues

**DOI:** 10.1186/s12864-015-2291-9

**Published:** 2016-01-15

**Authors:** Melanie A. Huntley, Melanie Lou, Leonard D. Goldstein, Michael Lawrence, Gerrit J.P. Dijkgraaf, Joshua S. Kaminker, Robert Gentleman

**Affiliations:** Department of Bioinformatics and Computational Biology, Genentech Inc., 1 DNA Way, South San Francisco, USA; Department of Molecular Oncology, Genentech Inc., 1 DNA Way, South San Francisco, USA

**Keywords:** RNA-DNA difference, RNA-editing, ADAR

## Abstract

**Background:**

RNA-editing is a tightly regulated, and essential cellular process for a properly functioning brain. Dysfunction of A-to-I RNA editing can have catastrophic effects, particularly in the central nervous system. Thus, understanding how the process of RNA-editing is regulated has important implications for human health. However, at present, very little is known about the regulation of editing across tissues, and individuals.

**Results:**

Here we present an analysis of RNA-editing patterns from 9 different tissues harvested from a single mouse. For comparison, we also analyzed data for 5 of these tissues harvested from 15 additional animals. We find that tissue specificity of editing largely reflects differential expression of substrate transcripts across tissues. We identified a surprising enrichment of editing in intronic regions of brain transcripts, that could account for previously reported higher levels of editing in brain. There exists a small but remarkable amount of editing which is tissue-specific, despite comparable expression levels of the edit site across multiple tissues. Expression levels of editing enzymes and their isoforms can explain some, but not all of this variation.

**Conclusions:**

Together, these data suggest a complex regulation of the RNA-editing process beyond transcript expression levels.

**Electronic supplementary material:**

The online version of this article (doi:10.1186/s12864-015-2291-9) contains supplementary material, which is available to authorized users.

## Background

RNA-editing is an enzyme-mediated post-transcriptional modification whereby individual nucleotide residues are altered in the nascent RNA transcript molecule [1]. In contrast to the traditional dogma of DNA makes RNA makes protein, RNA-editing is a non-heritable change to the RNA, which can result in both increased diversity of the transcriptional repertoire, and of the resulting peptide products.

The most common RNA-editing alteration is enzymatic deamination of adenosine nucleotides to inosine, by the family of ADAR enzymes [2, 3]. These are referred to as canonical A-to-I editing events. Since much of the cellular machinery treats inosine as a guanine nucleotide, A-to-I events are typically discovered and referred to as A-to-G in sequencing data.

There are 3 members of the ADAR family in vertebrates: Adar, Adarb1, and Adarb2 (also called Adar1, Adar2, and Adar3, respectively). A number of substrates are reported to be edited by Adar and Adarb1 [4–6]. However, there is no evidence to support the enzymatic activity of Adarb2 [7].

The gene for Adar produces two main protein isoforms. Expression of the longer Adar isoform, p150, is inducible by interferon stimulation [8] and has both nuclear and cytoplasmic localization [9, 10]. The shorter isoform of Adar, p110, is more constitutively expressed and nuclear localized [10]. It has been suggested that the nuclear Adar proteins edit pre-mRNAs prior to splicing, while the cytoplasmic p150 isoform may edit viral RNAs or microRNAs in the cytoplasm [4, 11–14].

Owing to the role of interferon in defense against microbial infection, the p150 isoform of Adar has been thought to function in the editing of double stranded viral RNA. In humans, double stranded RNA structures often form in the long 3’ UTR regions of genes when juxtaposed Alu repeat elements are present. These inverted transposable elements, which are remnants of ancient viral insertions, thus form the secondary structure substrate for hyper-editing [15].

In contrast to the hyper-editing of many adenosine residues in close proximity, site-selective editing tends to occur consistently at specific residues, and even within the coding sequence of transcripts. Aside from the propensity of RNA substrate to form hairpin structures, little is known about other mechanisms regulating this more selective form of editing. The importance of site-selective editing events is highlighted by the necessity of an Adarb1 mediated edit in the neurotransmitter receptor protein GluR-B for developmental viability in mice [16].

The ADAR gene family is highly conserved across metazoans, and highly expressed in the nervous system [4, 17, 18]. This pattern of evolutionary constraint suggests an important role of RNA-editing in the development and maintenance of the metazoan nervous system. RNA-editing deficiencies are observed in epilepsy, amyotrophic lateral sclerosis (ALS), Aicardi-Goutieres syndrome (AGS), schizophrenia, suicidal depression, and other neurodegenerative diseases [19–21].

RNA-editing has also been implicated in functions and disease outside the nervous system. Site-specific editing of AZIN1 is associated with more aggressive tumor-initiating potential in hepatocellular carcinoma [22], while an overall decrease in hyper-editing of Alu repeats has been found in brain, prostate, lung, kidney and testis tumors [23]. Analysis of RNA-seq data from The Cancer Genome Atlas (TCGA) demonstrated alterations in editing levels between tumors and matched normal tissues from the same patients [24, 25]. In some cancers, the editing level correlated with patient survival [24]. Cell viability assays for a small number of editing events that result in protein alterations were observed to significantly effect cell survival [25].

It is clear from these examples that RNA-editing is functionally important, and must require tight regulation to prevent dysfunction. However very little is known about how this regulation occurs. We do not have a thorough understanding of the variability in editing between individuals, nor the intra-individual variability across tissues to properly address this question. Furthermore, there has not yet been a genome-wide study to address, in an unbiased manner, the scope of functionally relevant RNA-editing in healthy individuals.

Previous high throughput RNA-editing surveys have been limited in the range of tissues studied, with a bias towards central nervous system (CNS) tissues, or in their genomic breadth. Transcriptome-wide RNA-seq analyses of RNA-editing in mice have largely been limited to whole brain and cerebral cortex [26, 27].

A valuable study of RNA-editing was performed across seven distinct tissues from a single human individual. However, the study used specific padlock probes, not transcriptome wide RNA-seq, and resulted in only 569 putative RNA-editing sites [28].

In order to investigate the mechanisms underlying the regulation of RNA-editing, we present an analysis of a novel transcriptome wide RNA-seq dataset from nine tissues in a single individual mouse. Additionally we present a comparison and combined analysis of 20 previously published RNA-seq samples from mice of the same strain across a variety of tissues. To the best of our knowledge, this is the only dataset for which we can address in a genome-wide fashion, questions both on the variability of RNA-editing across tissues in a single individual and within specific tissues across individuals. We then use these data to investigate the underlying mechanisms regulating the important process of RNA-editing.

## Results

### Collecting high confidence RNA-DNA differences

**Fig. 1:**
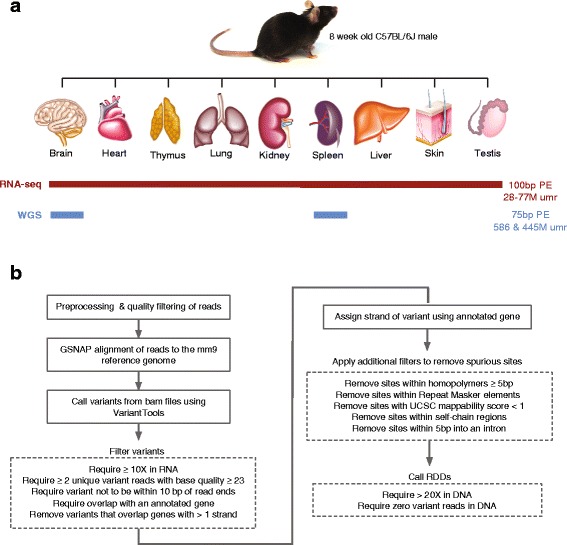
Experimental design (**a**) and sequencing processing workflow to call RNA-DNA differences (RDDs) (**b**). Tissues were harvested from an 8 week old male C57BL/6J mouse. RNA was isolated from 9 tissues and individually used for 100 bp paired-end RNA-sequencing. After alignment to the mm9 genome the number of uniquely mapped reads (umr) was between 28 million and 77 million for each tissue sample. DNA was extracted from the brain and spleen tissues, and sent for 75 bp paired end whole genome sequencing, to average depths of 31X and 23X coverage (586 and 445 million uniquely mapped reads, respectively)

We obtained a high confidence set of RNA-DNA differences (RDDs) by analyzing the RNA and DNA from various tissues of a single adult mouse (Fig. 1). In parallel, we processed 20 samples from 3 previously published datasets to serve as biological replicates for our samples. The previously published samples included RNA-seq from brain, cerebellum, heart, kidney, liver, and testis.

**Fig. 2:**
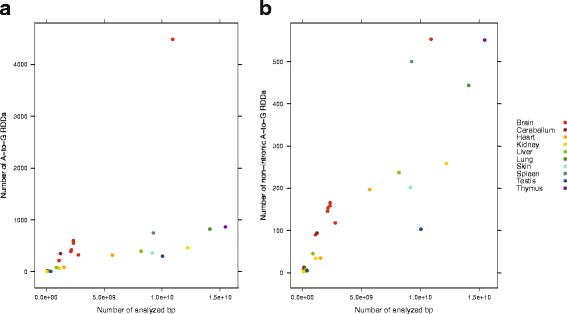
The total number of A-to-G RNA-DNA differences (RDDs) called in each sample, versus the number or analyzed base pairs (number of uniquely mapped reads × length of reads) (**a**). The nine samples with the largest number of analyzed base pairs correspond with the high coverage samples generated in this study. The lower coverage samples were derived from previously published datasets. The high coverage brain sample in this study is remarkable for having a higher amount of RDDs. However, when all RDDs that overlap an intron are discarded the high coverage brain sample is no longer a strong outlier compared to the other high coverage tissues (**b**)

We identified 13,798 RDDs within our dataset, including 5053 novel A-to-G changes not previously reported in the Darned or Radar databases of RNA-editing sites [29, 30]. In the non-brain samples we analyzed, the number of A-to-G RDDs detected increases with sequencing depth (Fig. 2a). This suggests that many sites are edited at a low frequency and the ability to detect these edits relies on sufficient sequencing depth. The greater sequencing depth of our samples, and the inclusion of tissues underrepresented in the mouse Darned and Radar databases likely explains the high number (80 %) of A-to-G changes we detect as novel in this study. Among the 9 high coverage samples that we generated, the brain has remarkably more RDDs than the other tissues, even after accounting for sequencing coverage. This is consistent with previous evidence that RNA-editing is frequently observed within transcripts of the central nervous system [31]. Interestingly, much of the RNA-editing within the brain tissue samples occurs within introns, rather than exons. Further inspection reveals that the transcripts derived from the brain samples have a higher proportion of reads that map to intronic rather than exonic gene regions and that this generally correlates with the proportion of genic RDDs within introns (Additional file 1: Figure S2 and Additional file 2: Figure S3). After removing RDDs that fall within introns, the brain samples are no longer the marked outlier for the number of observed RDD sites (Fig. 2b). Thus both the sequencing depth of coverage, and genic context of potential editing sites plays a large role in the ability to detect RNA editing events.

### Genic context of editing sites

**Fig. 3:**
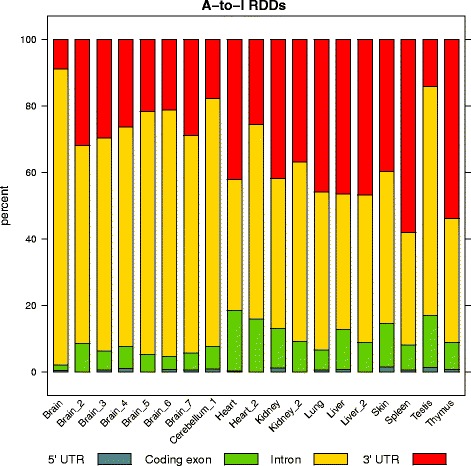
Relative proportions of where A-to-I RDDs are located within genes (5’ UTR, coding exons, introns, and 3’ UTR), across samples. The lower coverage samples derived from previously published datasets are denoted by an underscore and number following the tissue name. The percent of A-to-I RDDs that are intronic is consistently higher within brain samples, including the lower coverage brain samples

To investigate the functional relevance of the A-to-G RDDs identified from our analysis, we used RefSeq gene annotations to determine the genic context and predicted functional consequence (ie. synonymous, non-synonymous) of each RDD. Across all tissues, most of the RDD sites occurred within introns (Fig. 3). This is somewhat surprising since the standard poly-d(T) library prep for RNA-sequencing should enrich for mature mRNA transcripts whose introns have already been spliced out. Less than 5 *%* of A-to-I RDDs fall within coding exons, and only 159 would result in a non-synonymous change. Further inspection of the genic context of editing sites revealed consistent results across samples within the same tissue. For example, all eight brain tissue samples showed a much higher proportion of intronic RDDs to coding exon RDDs than any of the other tissues. Likewise, the two heart samples had the highest coding exon RDD to intronic RDD ratio. Therefore RNA editing preferentially occurs within intronic sequence, and the amount of intronic substrate available appears to be tissue dependent.

We next considered whether certain classes of genes were preferentially edited within our samples as this might reveal a regulatory aspect of the editing process. Within each tissue, we collected genes containing protein altering RDDs and tested for enrichment of any functional category amongst the genes normally expressed within that tissue. We performed a Gene Ontology analysis for each tissue individually, where the universe of genes was simply the set of genes with an RPKM expression level ≥ 10 within that tissue. Additional file 3: Table S1 reports the results of this analysis, filtering for odds ratios > 10, and at least 2 edited genes per category. Only the edited genes within brain and within testis show significant enrichment for any gene ontology. We find that genes involved in ionotropic glutamate receptor signalling are enriched for non-synonymous RDDs amongst all genes expressed within brain tissue. This result is observed in both the high and low coverage brain tissue samples. These sites are highly important for normal brain functioning and often have editing frequencies near 100 *%*. However, the same six edited genes (Gria2, Gria4, Grik2, Grik5, Fcho2, and Cadps) are found to recur in most of the significant GO categories for the brain samples. Likewise for the testis results, three edited genes (Nup153, Ddx25, and Plekha8) account for the results. Thus there are few ontologies enriched for protein altering RNA-editing, and these are driven by a very small number of edited genes.

### Variation in RNA-editing within and across individuals

**Fig. 4:**
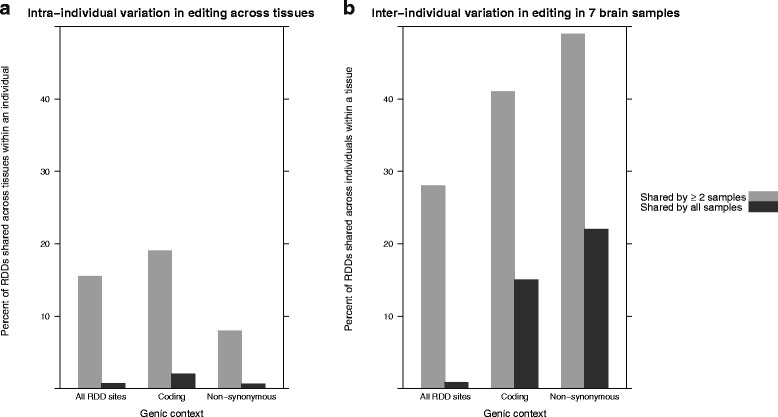
Variation in editing sites across 9 tissues within an individual (**a**). Inter-individual variation in editing within a tissue, across 7 individuals (**b**). An editing site was considered shared if it was called as an RDD in more than one sample, regardless of the editing frequency. RDD sites were further classified as coding and non-synonymous according to RefSeq gene annotations

After examining aggregated trends in RNA-editing across tissues and individuals, we next surveyed the variation in editing per site. This finer scale analysis is driven by the assumption that functionally relevant editing sites should be recurrently edited across individuals, and possibly across tissues. We first looked at the number of A-to-I RDD sites that were shared among the 9 tissue samples from our single male mouse (Fig. 4a). An editing site was considered shared if it was called as an RDD in more than one sample. Thus the frequency of editing at a given site was ignored, and the site was treated as a binary variable: edited (called as an RDD) or not edited (RDD was not called). Only 15.5 *%* of sites are shared between any two tissues, and even fewer (0.7 *%*) are shared across all 9 tissues. These numbers both increase slightly when only RDDs within coding exons are considered (19 % and 2 %, respectively). This might suggest that more functionaly relevant RDDs, such as those within coding regions, are more likely to be shared across tissues. However, for RDDs predicted to be non-synonymous, and thus more likely to cause a functional change in the resulting protein product, the proportion that are shared across tissues decreases to 8 %. Thus, the recurrence of editing sites across tissues is low, but increases modestly when considering only synonymous editing events within coding exons.

We next looked at the inter-individual variability of calling RDDs. If RDDs are functionally important, we assume that their presence will be consistent across individuals, when looking in the same type of tissue. Here we included the previously published RNA-seq samples (excluding the male samples from Brawand et al. (2011) due to insufficient sequencing depth), to serve as biological replicates for four of our tissues. We had 7 adult brain samples, and 2 samples each for the heart, kidney, and liver tissues.

Within the 7 brain samples we found a much higher proportion of RDD sites that were shared (by any two individuals, and by all individuals) than across tissues within a single individual (Fig. 4b). Thus there is more similarity in the editing of brain transcripts from different individuals than from different tissues within the same individual. We also observe an increase in the proportion of brain RDDs shared between individuals when we consider coding and then non-synonymous sites. Thus shared RDDs within brain transcripts, which we presume to be functionally relevant, appear enriched for non-synonymous sites.

We next wanted to see if this pattern held true in the other tissues. Since we only had 2 samples per tissue for the heart, kidney, and liver, for consistency we subset our brain samples to include only 2 samples (one from our generated data, and one from Brawand et al. (2011)). The brain samples had the highest consistency in RDD recurrence within coding and non-synonymous sites (Additional file 4: Figure S4). The other tissues had higher consistency than brain overall, but not for coding and non-synonymous sites. Additionally, except for kidney, there was more similarity in editing within a tissue from different individuals than across tissues. Thus, for the presumed most functionally relevant RDDs, we observe more differences between tissues than between individuals.

### Tissue specific RNA-editing

**Fig. 5:**
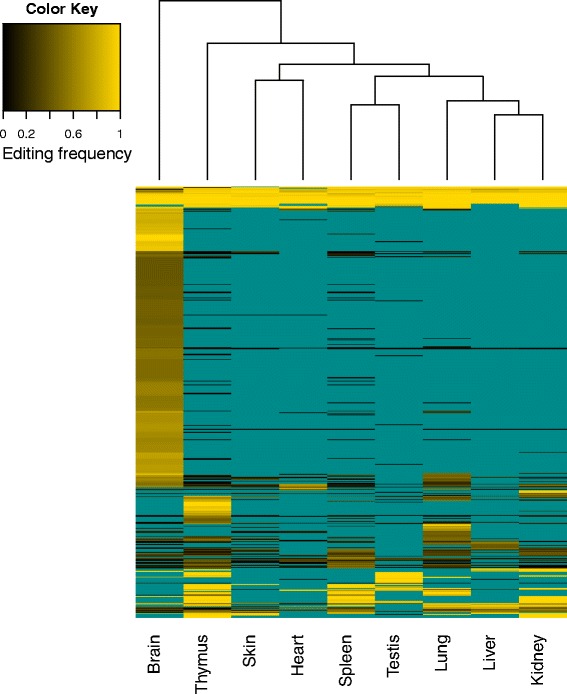
A-to-I editing frequency at 575 sites across nine tissues, represented as a heatmap. These sites were selected on the basis of having at least 10X coverage and an editing frequency > 0.4 in at least one tissue. Tissues for which the coverage was < 10X at a site appear as cyan, and these correspond to sites that would have been removed during the variant filtering process for calling RDDs. Very few sites are highly edited across all nine tissues. Most sites that are edited in only one tissue are often not expressed (insufficient read coverage) in the other tissues

Having established a baseline for the intra- and inter-individual variability of RDD sites, we then investigated the possible mechanisms by which we observe differences in editing across tissues. There are several possible hypotheses to explain differential or tissue specific RNA-editing. One hypothesis is that the transcript being edited is not available or at sufficient abundance to be detected in one sample versus another. A second hypothesis is that there is instead a difference in the editing enzymes between the samples. We tested the first hypothesis and found that in the majority of cases where we observed an RDD within one tissue specifically, and not the other tissues, this was simply a result of insufficient expression of the transcript in the other tissues (Fig. 5). This result held true even when the previously published samples were included (Additional file 5: Figure S5). Therefore, differential expression of the substrate transcript seems to be a main cause of tissue specific RNA-editing.

### ADAR expression and isoform usage

Interestingly, we did find some examples of RDDs for which the transcript was sufficiently expressed, but where the editing frequency varied across tissues. To examine what might be causing this variability in editing frequency across tissues, we used the RNA-seq data to investigate the expression and isoform usage of the ADAR enzymes. This provided us with the unique opportunity to study the quantitative effect of ADAR expression on RNA-editing.

**Fig. 6:**
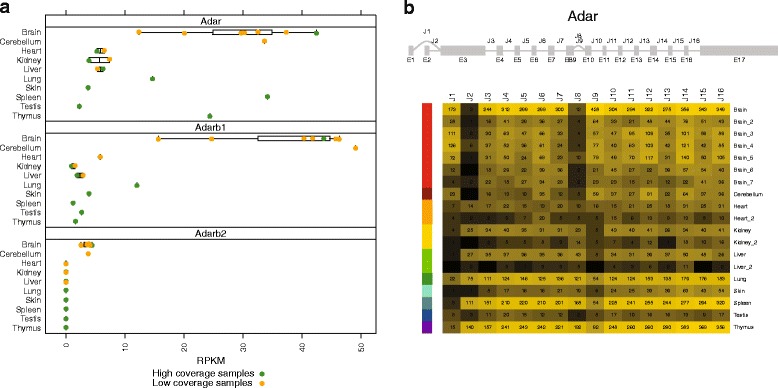
Expression level of Adar, Adarb1, and Adarb2 genes across tissues, as measured by RPKM (**a**). Splice junction usage across samples for ADAR transcripts (**b**). Raw counts of reads supporting each splice junction in each sample are included as text in the cells of the isoform heatmap. The heatmap color gradient represents the *l* *o* *g*2(*F* *P* *K* *M*+1) for each junction and scales from minimum (*black*) to maximum (*yellow*). ADAR is known to have a shorter, typically brain specific protein isoform (referred to as p110), and a more ubiquitously expressed longer protein isoform (p150). These two isoform variants are distinguished by the alternate 5’ UTRs that are used by the J1 and J2 mutually exclusive splicing events. J1 produces the shorter p110 isform while J2 produces the longer p150 isoform. Of note, an internal alternative splicing event, J8, tends to co-occur with the p150 isoform, while the J9 splice appears more frequently in samples with higher p110 isoform usage

There are three members of the Adar family of enzymes that are expressed within vertebrates: Adar, Adarb1, and Adarb2 (also called Adar1, Adar2, and Adar3, respectively). Our RNA-seq data provides us with both the overall expression level estimates for the Adars, and isoform level estimates based on splice junctions (Fig. 6).

Adar is most highly expressed in the brain and spleen samples, which is consistent with brain and spleen having higher number of observed RDDs after accounting for sequencing depth (see Fig. 2a). Adarb1 has the highest expression in brain, and seems to be more brain specific in its expression than Adar. However both Adar and Adarb1 have relatively modest expression in brain, with RPKMs lower than 50 in all samples. Adarb2 is exclusively expressed in the brain, however it is expressed at a very low level, and to date, there is no evidence supporting the editing activity of this enzyme [7]. Thus, Adarb1 and Adarb2 have nearly exclusive expression within brain tissue, while Adar has more appreciable expression across multiple tissues.

From the literature, it is expected that there are differences in Adar isoform usage across tissues, with the shorter p110 isoform more highly expressed in CNS tissues. Therefore, it is important to study gene expression at the isoform level as well. When we examined the data across our samples, we confirmed the elevated expression of the p110 isoform within brain samples (Fig. 6b). The longer p150 isoform was observed more frequently within the non-brain samples.

While spleen and thymus have Adar expression levels similar to those found in brain, the difference in isoform usage may result in differential regulation of editing between these tissues. There are some reported motifs prefered by Adar and Adarb1, but little is known about the specificity differences between the p110 and p150 isoforms of Adar. Both isoforms contain a z-alpha DNA binding domain at their N-terminal end, but the alternative 5’ UTR and splice of the p150 isoform encodes a second z-alpha domain upstream of the first. This extra z-alpha domain could potentially direct the editing enzymes to prefer a subset of nascent transcripts with proximity to a particular DNA sequence that interact with the z-alpha domains. Thus differences in the resulting protein isoforms may provide a mechanism for different selectivity of editing substrates.

We also discovered a correlation of the p110 (J1 junction) with an internal in-frame alternative splicing event (J9) that results in the addition of 26 amino acids between the second double stranded RNA-binding domain and the deamination domain. This region includes four potential phosphorylation sites. The functional effect of this change, in altering the relative position of the deamination domain to the bound RNA transcript, could play a role in the sequence context of RNA-editing events. Thus isoform usage may influence and correlate with specific motifs surrounding editing sites within a sample.

To test this hypothesis, we analyzed the neighboring nucleotides surrounding edited sites across tissues to look for tissue specific patterns that might elucidate editing specificity of the Adar isoforms (Additional file 6: Figure S6). We did not observe a strong difference in motifs surrounding edit sites across tissues. However, we did find that the genic context of the site appeared to have an effect, with sites in 3’ UTRs having the most consistent neighboring nucleotides across tissues.

### Regulation of differential editing

To test the hypothesis that editing enzyme expression levels might account for differences in editing frequency per site across tissues, we first needed a collection of sites that were sufficiently expressed across multiple tissues, and varibly edited. For this we required sites that had a minimum of 10X coverage in at least 2 samples, and for an RDD to have been called at the site within at least 1 sample.

**Fig. 7:**
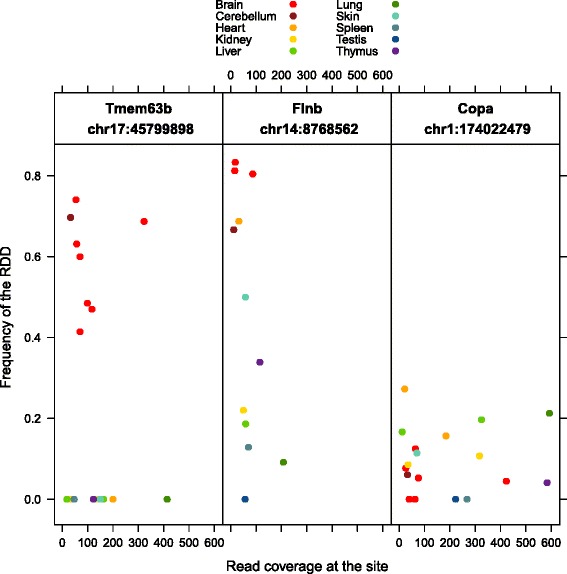
The variability in editing frequency per site across tissues. Three sites where the RDD encodes a non-synonymous change were selected and presented here (Tmem63b, Flnb, and Copa). Only samples with ≥ 10X coverage at these sites are plotted

We found 1831 such sites, 232 of which were within coding exons, and 136 were predicted to result in non-synonymous changes. Only 42 of these non-synonymous sites had sufficient coverage in more than half the samples. We highlight here three examples of non-synonymous RDD sites that are variably edited across multiple samples (Fig. 7).

We observed an RDD site in the gene Tmem63b which is expressed in all 9 tissues, but edited only within the brain samples. This site is found within the fifth coding exon upstream of the 3’ UTR, and results in a non-synonymous change from a glutamine to an arginine. The editing frequency at this site within brain samples ranges from 41 to 74 *%*. We also observed this brain specific pattern of editing in the human Tmem63b gene, using the human Illumina Body Map 2 RNA-seq dataset (NCBI GEO accession GSE30611, Additional file 7: Figure S7).

The gene Flnb contains a previously reported non-synonymous RDD [26]. The authors investigated RNA-editing within mouse brain tissue, and these samples are included in our analysis. We also observe RNA-editing at this site within our brain samples, but additionally observe a wide range of editing frequencies across tissues. In our samples, this site is most highly edited within brain and heart tissue, and this pattern is also found within the human Illumina Body Map 2 dataset (Additional file 7: Figure S7).

We also observed a non-synonymous RDD in the gene Copa. This site was previously unreported in mouse, but has been studied in human. This RDD has a much lower editing frequency, with the highest being 27 *%*, in one of the heart samples. This variably edited RDD site was one of the very few for which brain did not have the highest editing frequency. Curiously, the pattern of editing frequency across our mouse tissues was not consistent with that observed in the human Illumina Body Map 2 dataset (Additional file 7: Figure S7).

Having identified a collection of variably edited sites, we then tested the hypothesis that editing enzyme expression levels could explain the variations in editing across samples. To do this we calculated RPKM expression levels for the Adar genes (across coding exons), and RPKM estimates for the mutually exclusive 5‘ exons that distinguish the p110 and p150 isoforms of Adar. These expression levels were used to look for correlations with per site editing frequencies across samples.

**Fig. 8:**
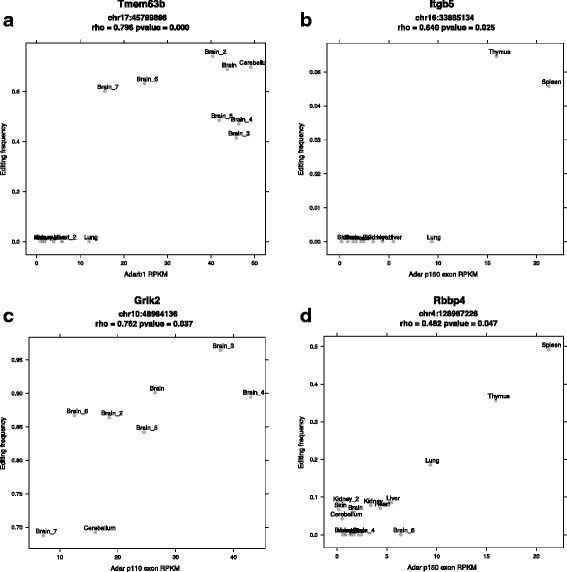
The correlation of editing frequency per site across samples with editing enzyme expression. Binary (edited vs. not edited) correlation patterns were observed for the non-synonymous edits in Tmem63b and Itgb5 with Adarb1 and Adar p150, respectively (**a**, **b**). Continuous correlation patterns were observed for the exonic editing sites in Grik2 and Rbbp4 with Adar p110 and Adar p150, respectively (**c**, **d**). Only samples with ≥ 10X coverage at these sites are plotted

Within coding exons we found 16 RDD sites whose variable editing frequencies had significant positive correlations with at least one of the editing enzymes or isoforms tested. After multiple testing correction the editing site within the gene Tmem63b remains significant. While the other 15 sites are not statistically significant after correcting for multiple testing, interesting trends were observed. Two distinct patterns may explain the regulation of editing (Fig. 8). The first is a binary pattern where editing appears to be on or off at a given site. In this situation, there is likely a threshold for the editing enzyme which determines if the substrate becomes edited or not. The second pattern is continuous rather than binary. In these cases, the editing frequency increases with the expression of the editing enzyme. Thus we are able to detect a small number of non-synonymous RDDs where the expression of the editing enzyme or isoform trends with the editing level of a ubiquitously expressed substrate transcript.

## Discussion

Previous studies suggest that RNA-editing is prevalent within transcripts of the central nervous system. In fact, the most well known A-to-I editing sites occur within coding regions of neuronal signalling genes. Among these, the transcripts of several genes involved with ionotropic glutamate signalling are typically edited at 100 % frequency. This begs the question of why these sites are not simply changed in the genome rather than edited in the transcript. Several studies report the importance of these editing events in modulating receptor activity in a developmental manner [11, 32–36] and provide a rationale for why these changes are not simply genomically encoded instead. This highlights an advantage of regulating transcriptome diversity via RNA-editing.

Here we have quantified that the number of sites which undergo A-to-I editing is remarkably higher in transcripts derived from the brain. While the ability to detect editing events increases with sequencing depth [22, 37], we continue to observe the brain transcriptome as an outlier even after accounting for this difference. We also confirm previous findings [38, 39] that the average editing frequency per site is elevated in brain tissues. These findings are now consistent across humans, non-human primates, and mice, suggesting a conserved role for RNA-editing in the neural transcriptome.

Interestingly, in our study, the vast majority of edited sites within brain samples occurred in intronic regions of genes. This enrichment for intronic editing accounted for the abnormally high amount of editing sites in brain tissues. The abundance of editing events within intronic sequence is intriguing as we did not expect high levels of sequencing coverage over introns. The RNA-sequencing library prep used in this study was a standard poly-d(T) protocol which should enrich for fully mature, poly-adenlyated mRNA transcripts that have already undergone splicing. However, intron retention appears to be a more common phenomenon within neural transcripts [40]. The recently reported phenomenon of recursive splicing in vertebrates [41] may result in slower processing of introns within the brain, and this may explain the abundance of intronic sequence we observe within our brain tissue data.

Previous studies have found that RNA editing in mice occurs less frequently than in humans [42]. This is thought to be related to hyper editing of Alu repeat elements within the 3’ UTRs of human transcripts. Alu repeats are an active primate specific SINE retrotransposon and are abundant within the human genome. When Alu sequences are juxtaposed within transcripts, they can form a double-stranded RNA hairpin loop structure which is an ideal substrate for ADAR enzymes. Mice do not have Alu repeats, but they do have four related active SINEs (B1, B2, 1D, and B4). The higher divergence between these mouse repeats is thought to result in lower editing levels than those observed within the Alu elements of humans, as fewer double stranded hairpin loops are expected to form within mouse transcripts. However, we do still observe a high proportion of editing sites within the 3’ UTR of transcripts. In spleen and thymus, the majority of the editing occurs within these exons. Therefore, despite lower levels of editing in mouse samples, the distribution of edited sites is largely consistent between humans and mice.

RNA-editing patterns varied across all tissues examined and sites edited in multiple tissues were not common. We demonstrate that tissue specific editing is largely a consequence of the tissue specific expression of the edited transcript. While our data do not argue against the existence of tissue specific editing cofactors, it appears that a major regulator of editing is the expression of the edited transcripts.

However, we did find evidence for a small number of transcripts expressed in multiple tissues but with variable levels of editing. In several of these cases a change in the expression or isoform usage of the editing enzymes correlates with the observed difference in editing. This suggests that the editing frequency is regulated by expression of a particular editing enzyme. In fact, the editing sites in both Flnb and Copa have been previously studied in the context of human hepatocellular carcinoma [43]. Using both tumor and cell line over-expression and knock down experimental data the authors present a compelling case for Adarb1 levels regulating editing in both genes. While they did not investigate the p150 isoform of Adar, they found that the p110 isoform enhanced editing in Flnb, but not in Copa.

While the gene Tmem63b is expressed in multiple tissues, editing is only observed within brain. This simpler, binary editing status is consistent with the brain having the highest expression of both Adarb1 and nearly exclusive expression of the Adar p110 isoform. However, Adars can dimerize to form homo- and heterodimers [44–49]. This combinatorial effect can add yet another layer of complexity to the regulation of editing, making even the simpler cases of differential editing, such as Tmem63b, less straightforward to explain. After accounting for substrate expression, we believe that the editing enzyme expression and isoform usage plays an important role in regulating the observed differences in the amount and location of editing across tissues and cell types.

## Conclusions

This study within mouse tissues is largely consistent with genome wide patterns of editing described in human datasets [38], and further explains how RNA-editing differences can be differentially regulated. The recurrence in editing patterns within tissues, across individuals, and even species, reinforces RNA-editing as functionally important for organismal development, survival, and evolution. Future studies testing the specificity of Adar isoforms, homo- and hetero-dimer combinations, and enzyme or substrate saturation are needed to further elucidate the regulatory mechanisms of this important cellular process.

## Methods

### Isolation of DNA and RNA from mouse tissues

An 8 week old C57BL/6J male mouse was obtained from The Jackson Laboratory and housed according to the animal use guidelines of Genentech, conforming to California State legal and ethical practices. This animal was euthanized by exsanguination with phosphate buffered saline (PBS) and tissues were collected, flash frozen and stored at –80 °C until further processing. DNA and RNA were extracted with either the All Prep DNA/RNA mini kit (kidney, liver, lung, spleen, testis and thymus), the RNeasy Fibrous tissue mini kit (heart and skin) or the RNeasy Lipid tissue mini kit (brain) according to manufacturers protocol (Qiagen).

### Sequencing data

We generated 100 bp, paired-end standard RNA-seq libraries from the 9 tissues (brain, skin, heart, liver, thymus, testis, spleen, lung and kidney) of the single adult male mouse described above. Each RNA-seq library resulted in 28 million to 77 million uniquely mapped reads.

Additionally, from this same mouse, we extracted genomic DNA and generated 75 bp paired end whole genome sequence libraries from the brain and spleen tissue. These produced 586 million and 445 million uniquely mapped reads, respectively.

For biological replicates of our dataset from a single mouse, we analyzed previously published RNA-seq data from 3 relevant datasets (GSE30352, GSE39866, and ERP000614), totalling 20 additional samples from 15 animals. The first dataset included 76 bp single-end RNA-seq data from 6 tissues (brain, cerebellum, heart, kidney, liver, and testis) of adult C57BL/6 mice [50]. This experiment used 2 separate mice for each tissue (one female, one male), with the exception of the testis tissue which only had the single male sample. In this dataset, only the female samples were sequenced to moderate depth (11 million to 20 million uniquely aligned reads), while the male samples had low coverage (1 million to 5 million uniquely aligned reads).

The second dataset included 80 bp single-end RNA-seq data from the cerebral cortex of embryonic (E17) and adult (3 - 4 months old) C57BL/6J female mice [27]. This experiment included 4 embryonic samples (resulting in 25 million to 29 million uniquely aligned reads) and 3 adult samples (with 26 million to 29 million uniquely mapped reads).

Finally, the third dataset included whole brain derived 76 bp paired-end RNA-seq data from two adult (ages 2 – 3 months) C57BL/6NJ female mice [26]. These data resulted in 15 million and 18 million uniquely mapped reads.

### Read processing and mapping

Our nine RNA-seq samples derived from a single individual mouse, and two whole genome DNA samples were sequenced on an Illumina HiSeq machine. The fastq sequence files for all RNA-seq samples, including the 20 previously published samples, were then filtered for read quality (keeping reads where at least 70 *%* of the cycles had Phred scores ≥ 23), and ribosomal RNA contamination. The remaining reads were then aligned to the mouse reference genome (mm9) using the GSNAP alignment tool [51]. Alignments were produced using the following GSNAP parameters: “-M 2 -n 10 -B 2 -i 1 -N 1 -w 200000 -E 1 –pairmax-rna =200000 –clip-overlap”. These steps, and the downstream processing of the resulting alignments to obtain read counts and RPKMs per gene (over coding exons of RefSeq gene models) are implemented in the Bioconductor package, HTSeqGenie (v 3.12.0) [52]. Only uniquely mapped reads were used for further analysis.

Our two DNA samples were processed similarly for quality, and aligned with the following GSNAP parameters specific for whole genome sequence: “-M 2 -n 10 -B 2 -i 1 –pairmax-dna =1000 –terminal-threshold =1000 –gmap-mode =none –clip-overlap”.

### Calling RNA variants

Single nucleotide variants (SNVs) were first called using the Bioconductor package VariantTools [53]. To increase our confidence in identifying true positive RNA-editing events, we required a mimimum of 10X coverage, at least two unique reads supporting the edited variant and base quality scores of 23 or higher. We discarded variants found solely within the 10 bp at either end of reads, due to the higher proportion of mismatches which tend to occur within these regions.

To infer the strand of a transcript, we considered only RNA-editing events that occurred within annotated genes, and discarded SNVs that were located in more than one gene on opposite strands.

To exclude spurious variants arising from regions of the genome that are problematic for short read alignment, we also discarded SNVs within homopolymer tracts, UCSC repeat masker track elements (low complexity, simple repeat, and satellites), UCSC multi-mappability regions, and self-chain regions (Additional file 8: Figure S1).

For added stringency, we also removed any intronic SNV that was within 5 bp of an exon-intron boundary. Although this was a very small fraction of the total number of SNVs, many were spurious alignments of reads into the intron with only a few basepairs originating from the downstream exon.

### Identification of RNA-DNA differences

A collection of high confidence RNA-DNA differences (RDDs) was then produced by stringent filtering of the SNVs from RNA using the whole genome sequence data. After all the filtering above, an SNV in the RNA was considered an RDD if the genomic data showed zero reads supporting the edited base, and had > 20X total coverage at the site in question. Although highly stringent, this gave us confidence in calling the remaining RDD sites RNA-editing events (Additional file 9: Figure S9).

We then further selected RDDs that represented the ADAR mediated A-to-I editing events (A-to-G or T-to-C changes for genes on the positive or negative strand, respectively).

## Availability

RNA-seq data generated for this study are available from NCBI’s Gene Expression Omnibus (GSE74747). Whole genome sequence data generated for this study are available from the European Nucleotide Archive (ERP010840).

## Additional files

Additional file 1
**Figure S2.** The ratio of reads mapping to introns versus exons in each sample. (PDF 5 kb)

Additional file 2
**Figure S3.** The percent of genic RDDs within introns versus the percent of genic reads mapped to introns. The proportion of intronic RDDs generally increases with increasing intronic read coverage, with brain and cerebellum samples having the highest of both. Samples are color coded by tissue. The lower coverage samples derived from previously published datasets are denoted by and underscore and number following the tissue name. (PDF 5 kb)

Additional file 3
**Table S1.** Gene Ontology analysis of non-synonymous A-to-I editing. (PDF 12 kb)

Additional file 4
**Figure S4.** Inter-individual variation in editing across 4 tissues. An editing site was considered shared if it was called an RDD in both individuals studied for a given tissue. RDD sites were further classified as coding and non-synonymous according to RefSeq gene annotations. (PDF 4 kb)

Additional file 5
**Figure S5.** A-to-I editing frequency at 575 sites across nineteen tissue samples, represented as a heatmap. These sites were selected on the basis of having at least 10X coverage and an editing frequency >0.4 in at least one tissue. Tissues for which the coverage was < 10X at a site appear as cyan. Very few sites are highly edited across all nine tissues. Most sites that are edited in only one tissue are often not expressed (insufficient read coverage) in the other tissues. (PDF 373 kb)

Additional file 6
**Figure S6.** Sequence motifs (produced by the motifStack R package) surrounding editing sites, separated by tissue and genic context. (PDF 2389 kb)

Additional file 7
**Figure S7.** The variability in editing frequency per site across tissues in the human Illumina Body Map 2 dataset. Only samples with ≥ 10X coverage at these sites are plotted. Three sites where the RDD encodes a non-synonymous change were selected and presented here (Tmem63b, Flnb, and Copa). (PDF 5 kb)

Additional file 8
**Figure S1.** Each of the five filters was applied independently and results plotted. The final column shows the results of all five filters applied sequentially. (PDF 4 kb)

Additional file 9
**Figure S9.** After filtering, the breakdown of observed RDDs by type and strand. (PDF 4 kb)
