# Supplementary figures and images for: Complex regulation of ADAR-mediated RNA-editing across tissues
Source: BMC Genomics. 2016 Jan 15;17:61. doi: 10.1186/s12864-015-2291-9 (PMC4714477; doi:10.1186/s12864-015-2291-9)

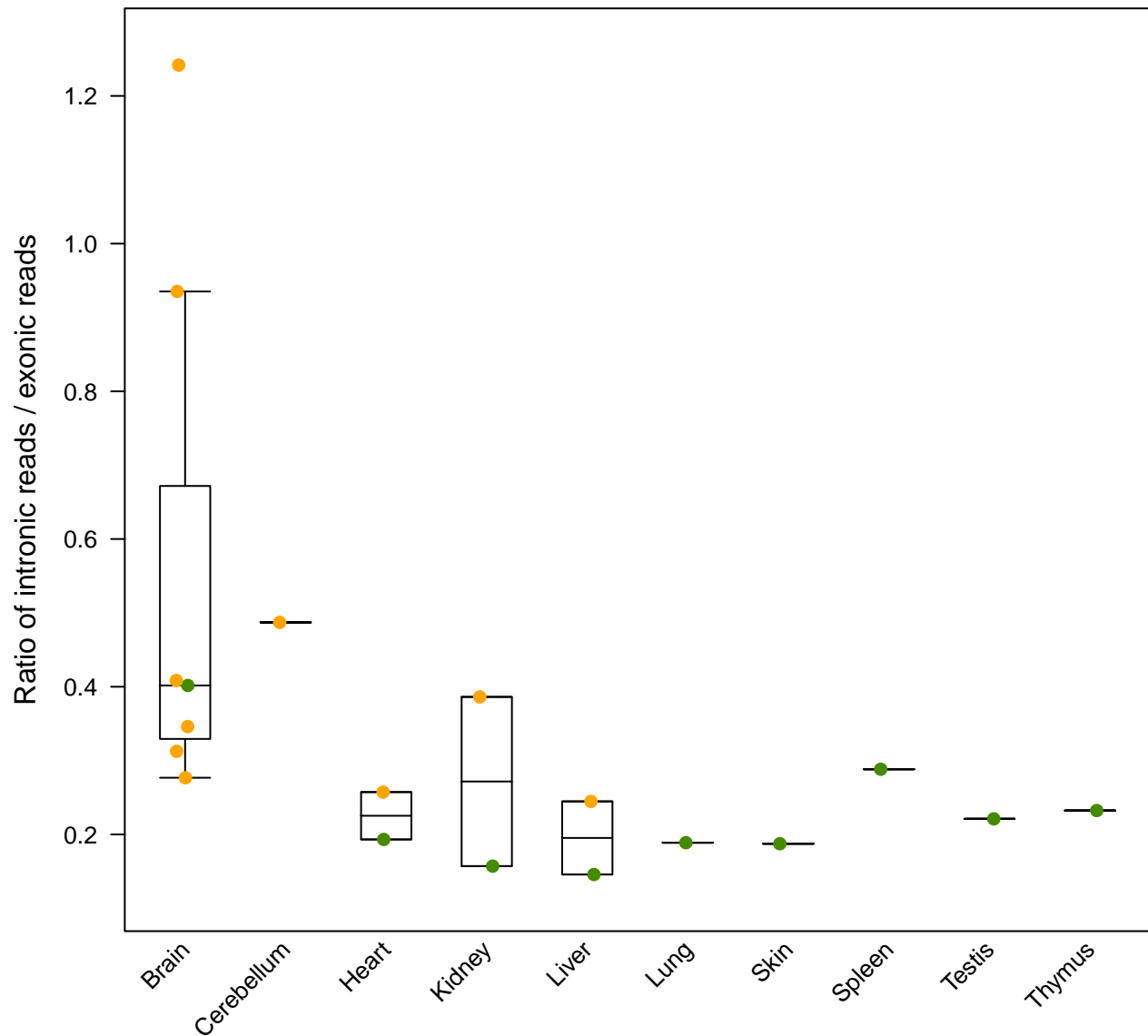

High coverage samples ●  
Low coverage samples ●

Supplement: Additional file 1 — Figure S2. The ratio of reads mapping to introns versus exons in each sample. (PDF 5 kb) [file 12864_2015_2291_MOESM1_ESM.pdf]

Percent of genic RDDs within introns

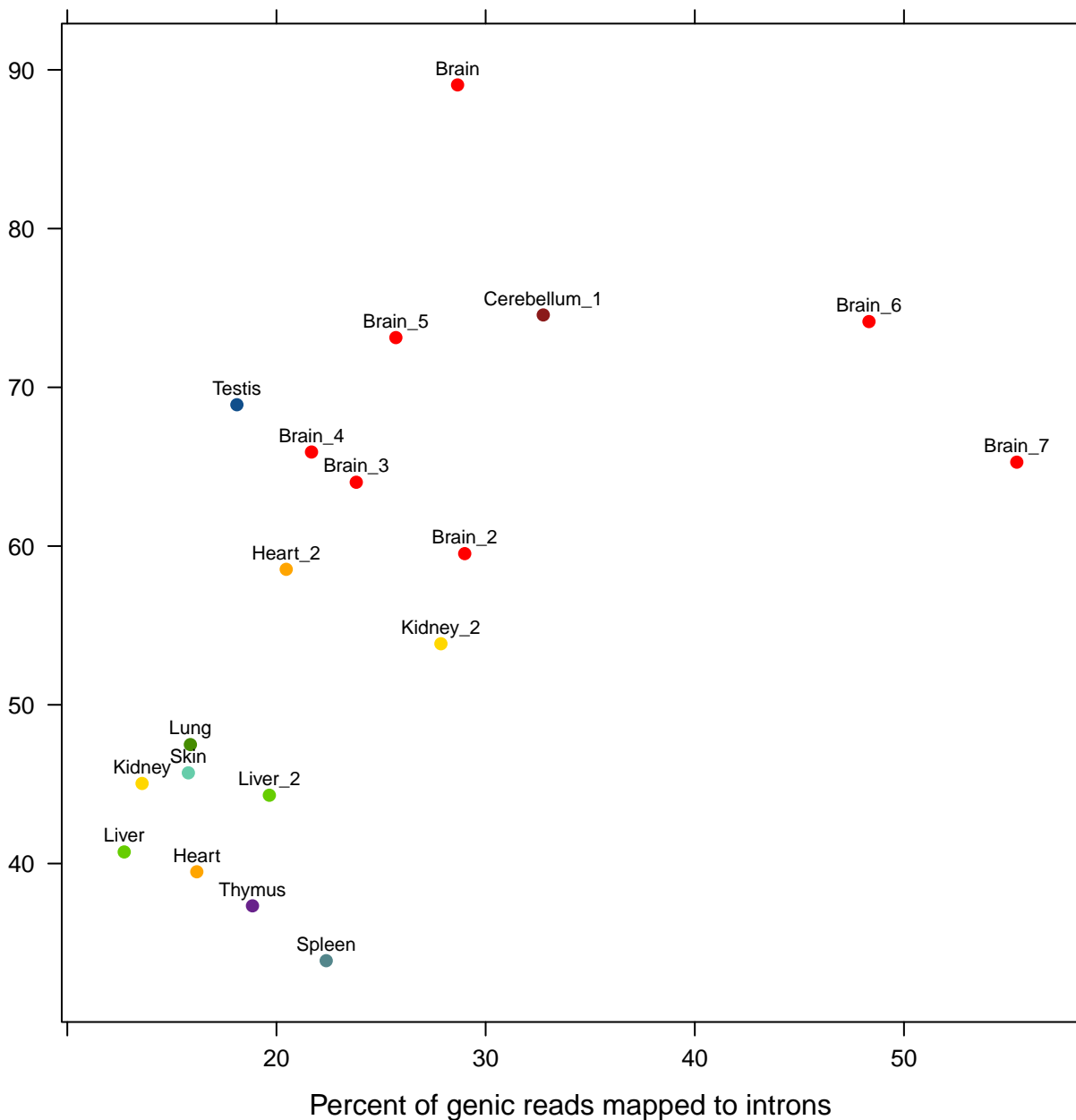

Supplement: Additional file 2 — Figure S3. The percent of genic RDDs within introns versus the percent of genic reads mapped to introns. The proportion of intronic RDDs generally increases with increasing intronic read coverage, with brain and cerebellum samples having the highest of both. Samples are color coded by tissue. The lower coverage samples derived from previously published datasets are denoted by and underscore and number following the tissue name. (PDF 5 kb) [file 12864_2015_2291_MOESM2_ESM.pdf]

## Inter-individual variation in editing

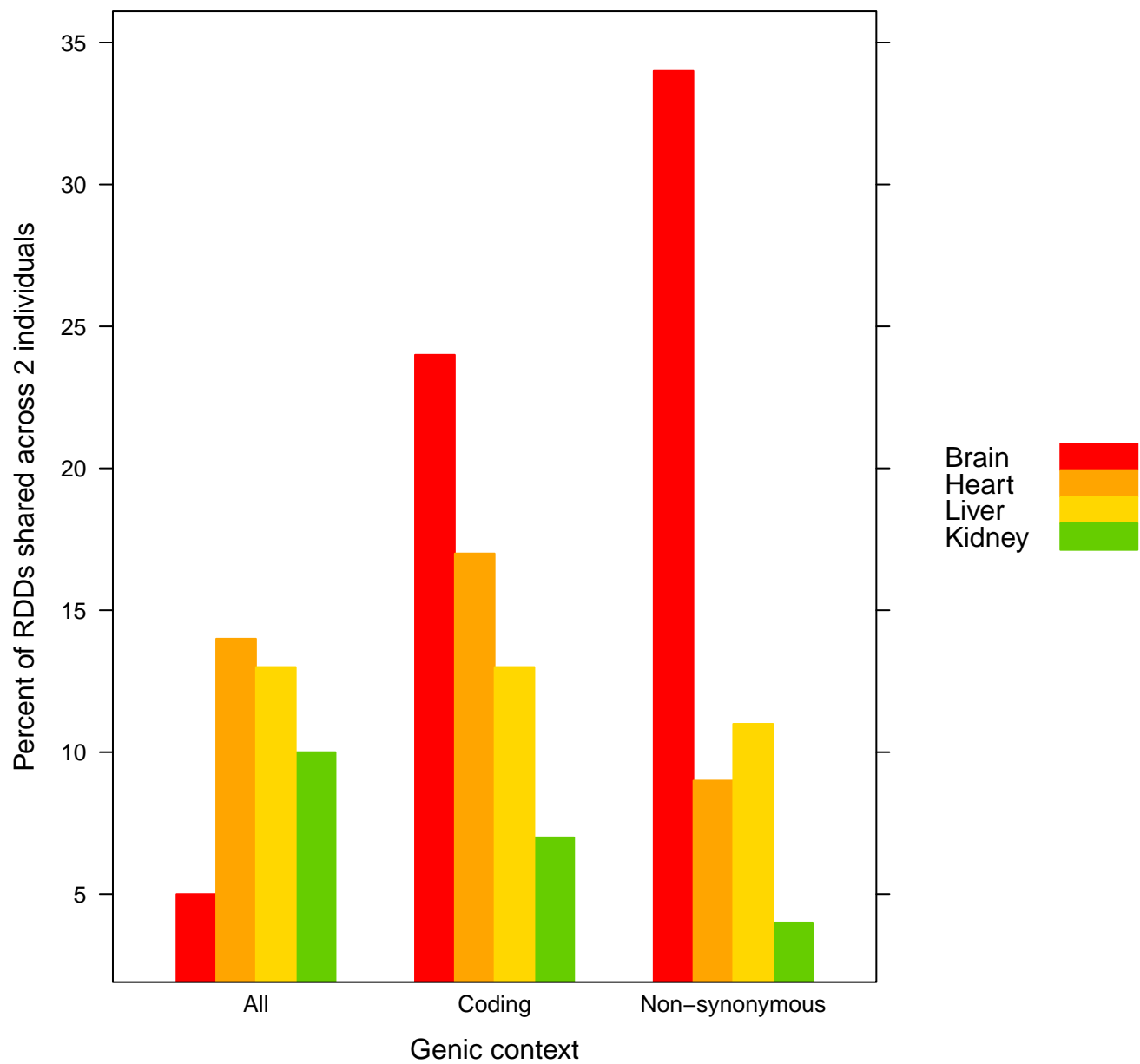

Supplement: Additional file 4 — Figure S4. Inter-individual variation in editing across 4 tissues. An editing site was considered shared if it was called an RDD in both individuals studied for a given tissue. RDD sites were further classified as coding and non-synonymous according to RefSeq gene annotations. (PDF 4 kb) [file 12864_2015_2291_MOESM4_ESM.pdf]

Color Key

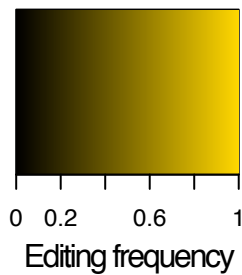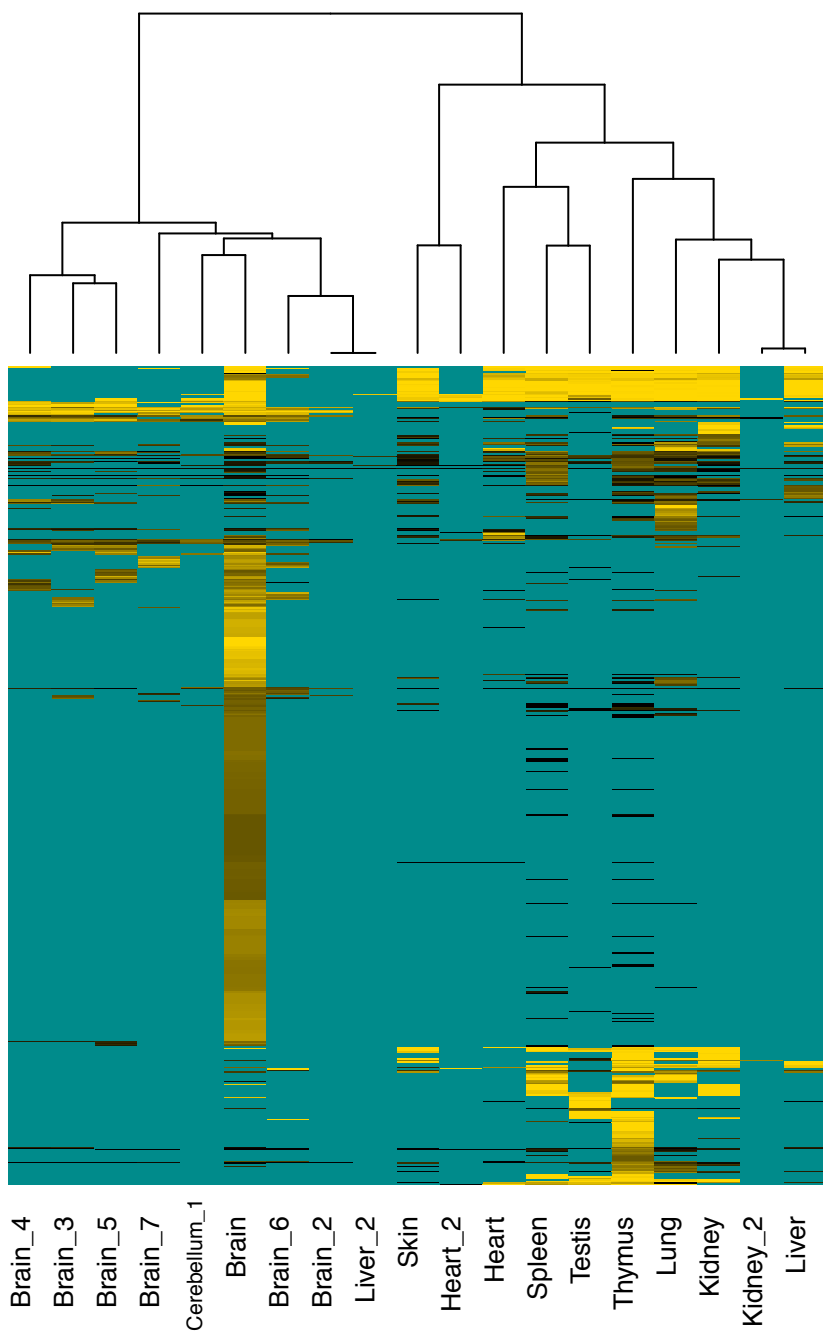

Supplement: Additional file 5 — Figure S5. A-to-I editing frequency at 575 sites across nineteen tissue samples, represented as a heatmap. These sites were selected on the basis of having at least 10X coverage and an editing frequency >0.4 in at least one tissue. Tissues for which the coverage was < 10X at a site appear as cyan. Very few sites are highly edited across all nine tissues. Most sites that are edited in only one tissue are often not expressed (insufficient read coverage) in the other tissues. (PDF 373 kb) [file 12864_2015_2291_MOESM5_ESM.pdf]

Brain     ●  
Heart    ●  
Kidney   ●     Liver    ●  
                 Lung    ●  
                 Testes ●

50     100     150

**Tmem63b**  
**chr6:44120349**

**Flnb**  
**chr3:58141802**

**Copa**  
**chr1:160302244**

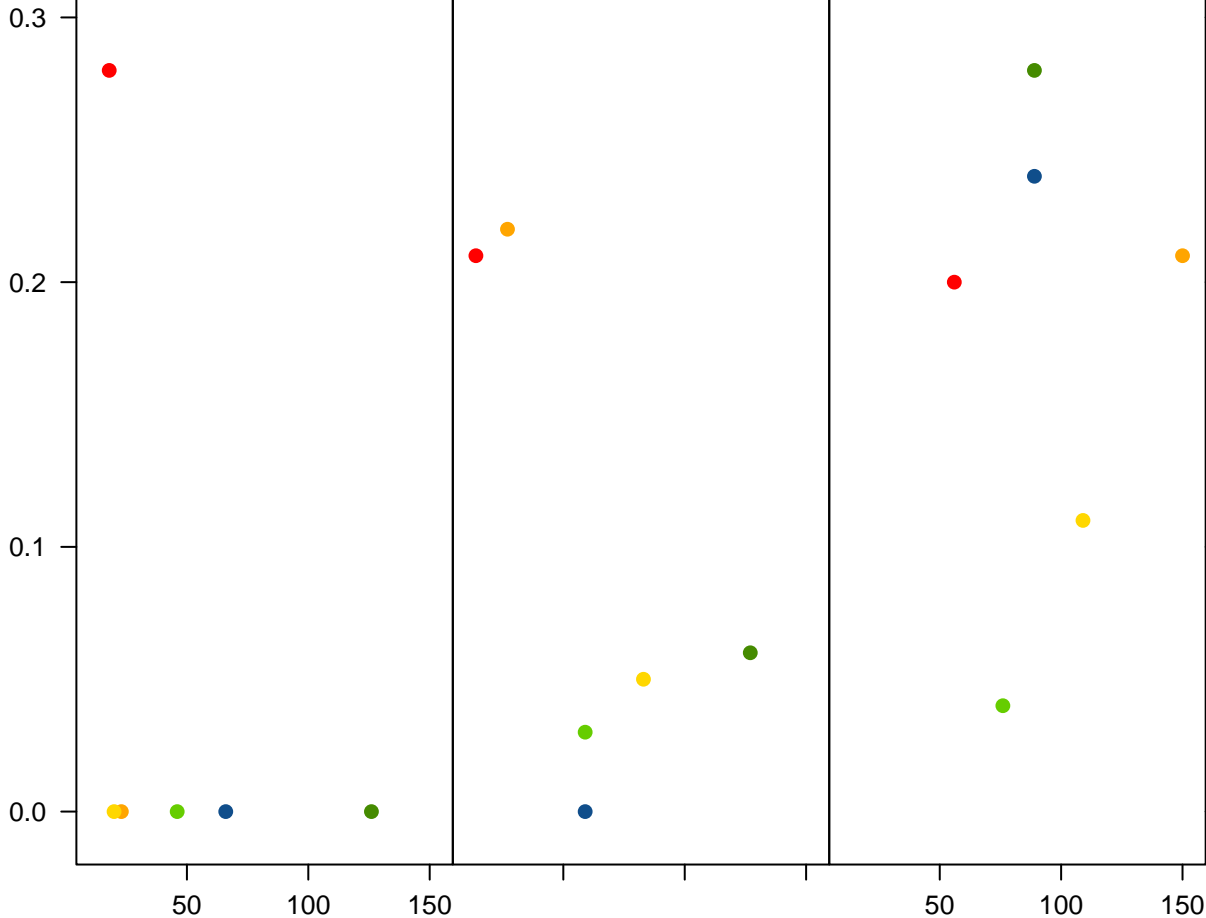

Supplement: Additional file 7 — Figure S7. The variability in editing frequency per site across tissues in the human Illumina Body Map 2 dataset. Only samples with ≥ 10X coverage at these sites are plotted. Three sites where the RDD encodes a non-synonymous change were selected and presented here (Tmem63b, Flnb, and Copa). (PDF 5 kb) [file 12864_2015_2291_MOESM7_ESM.pdf]

# RNA-DNA differences with various filters applied

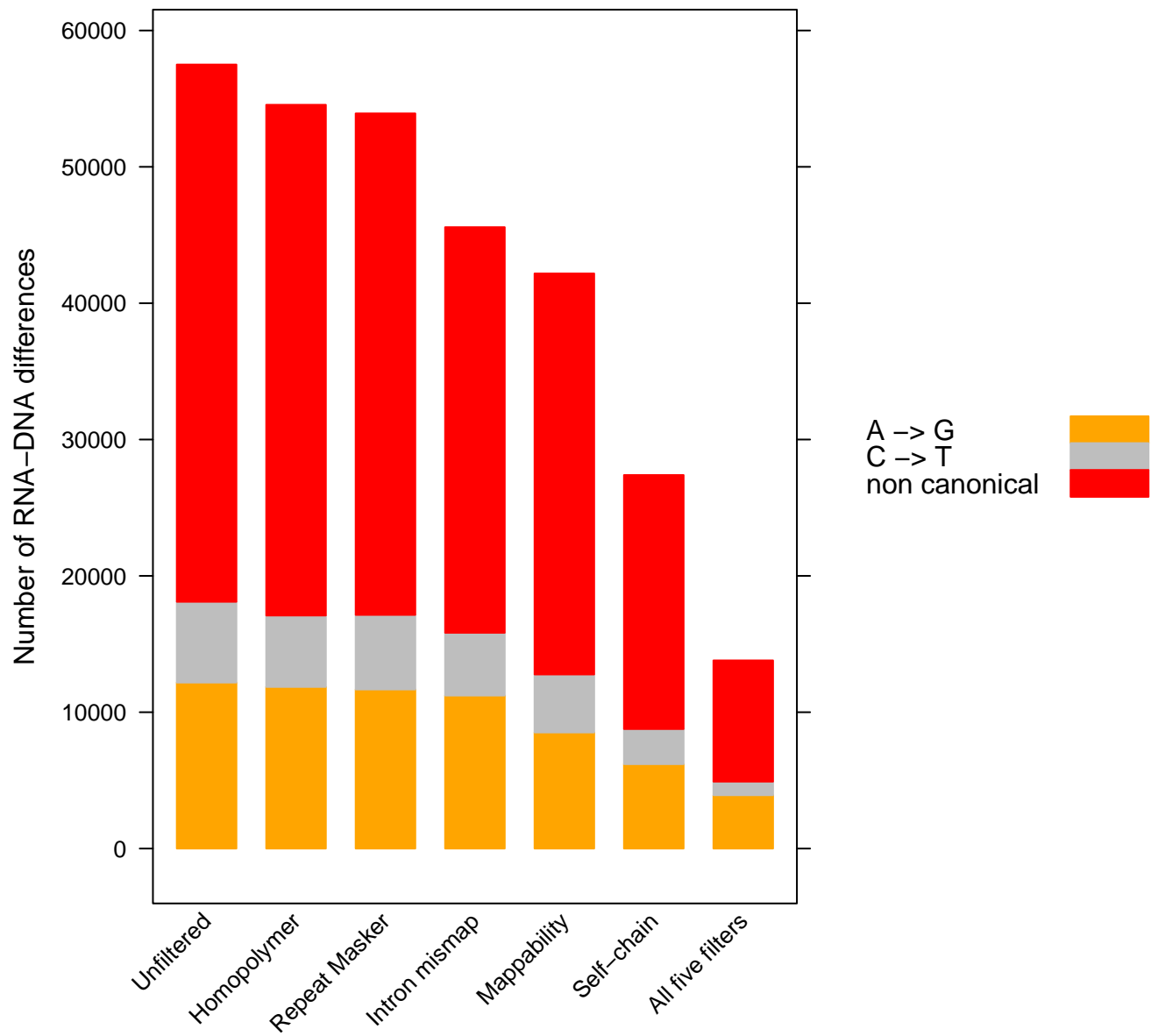

Supplement: Additional file 8 — Figure S1. Each of the five filters was applied independently and results plotted. The final column shows the results of all five filters applied sequentially. (PDF 4 kb) [file 12864_2015_2291_MOESM8_ESM.pdf]

**A**

**Tmem63b**  
 $\rho = 0.679$  pvalue = 0.002

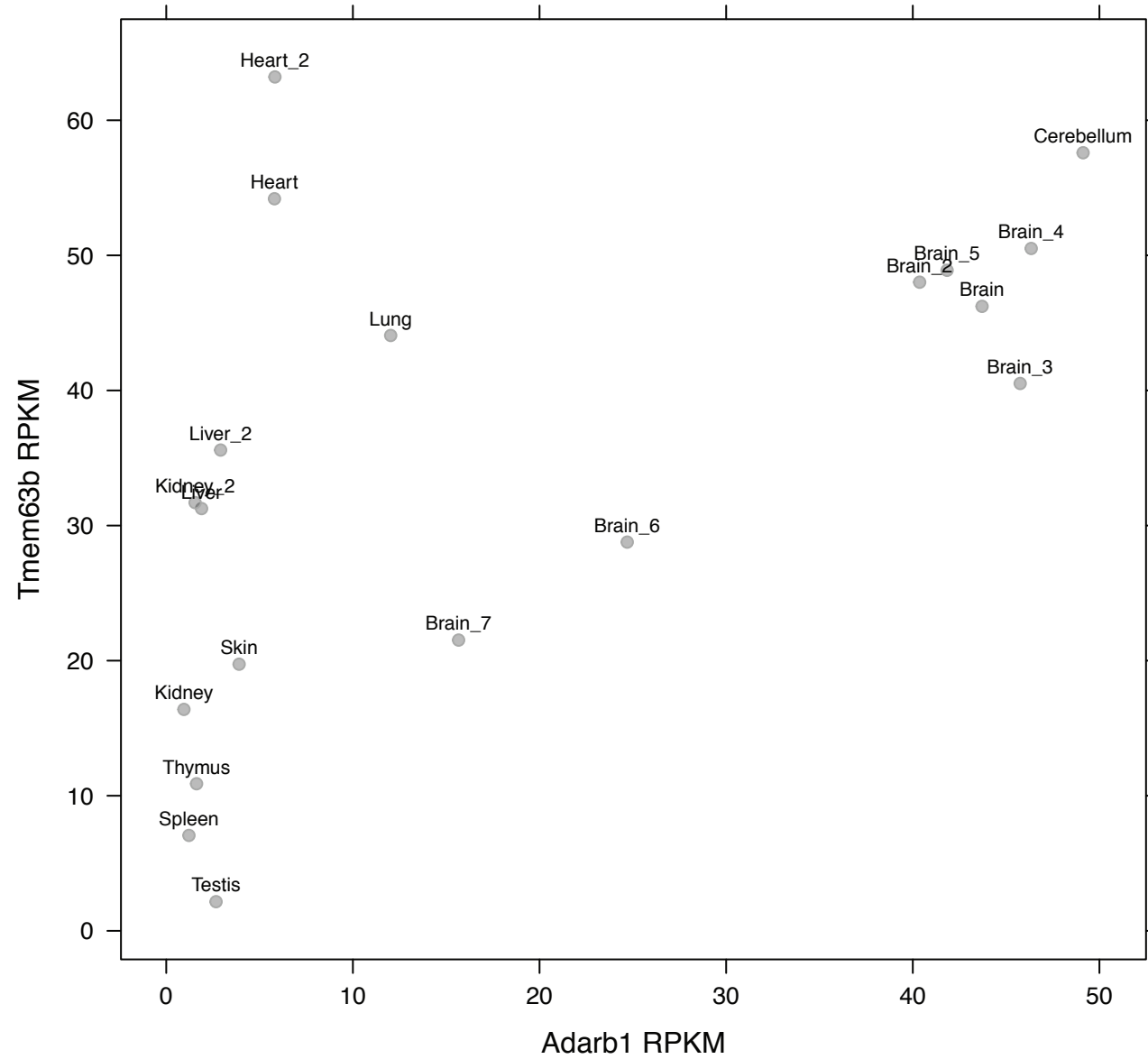**B**

**Itgb5**  
 $\rho = -0.289$  pvalue = 0.229

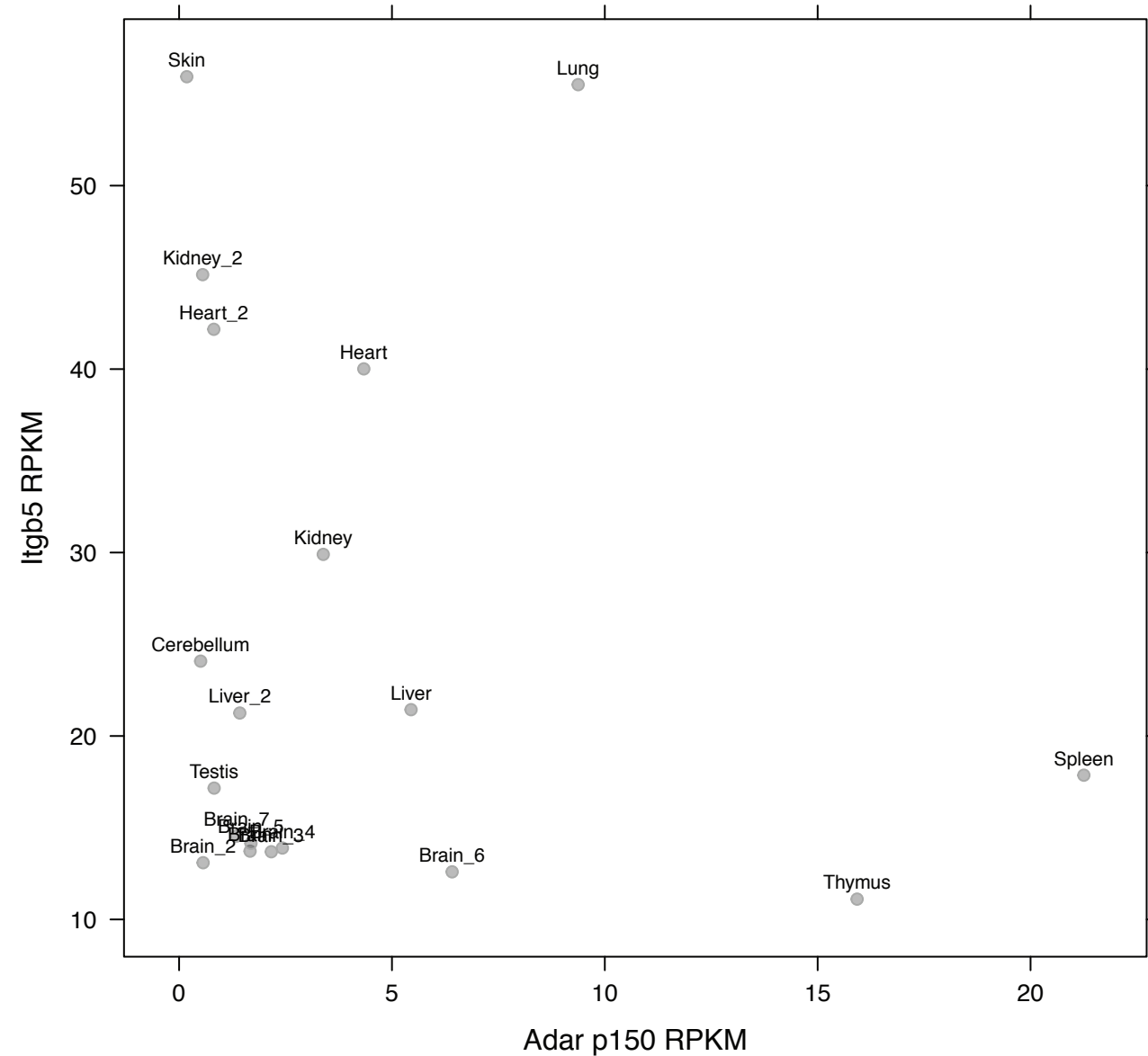**C**

**Grik2**  
 $\rho = 0.873$  pvalue = 0.000

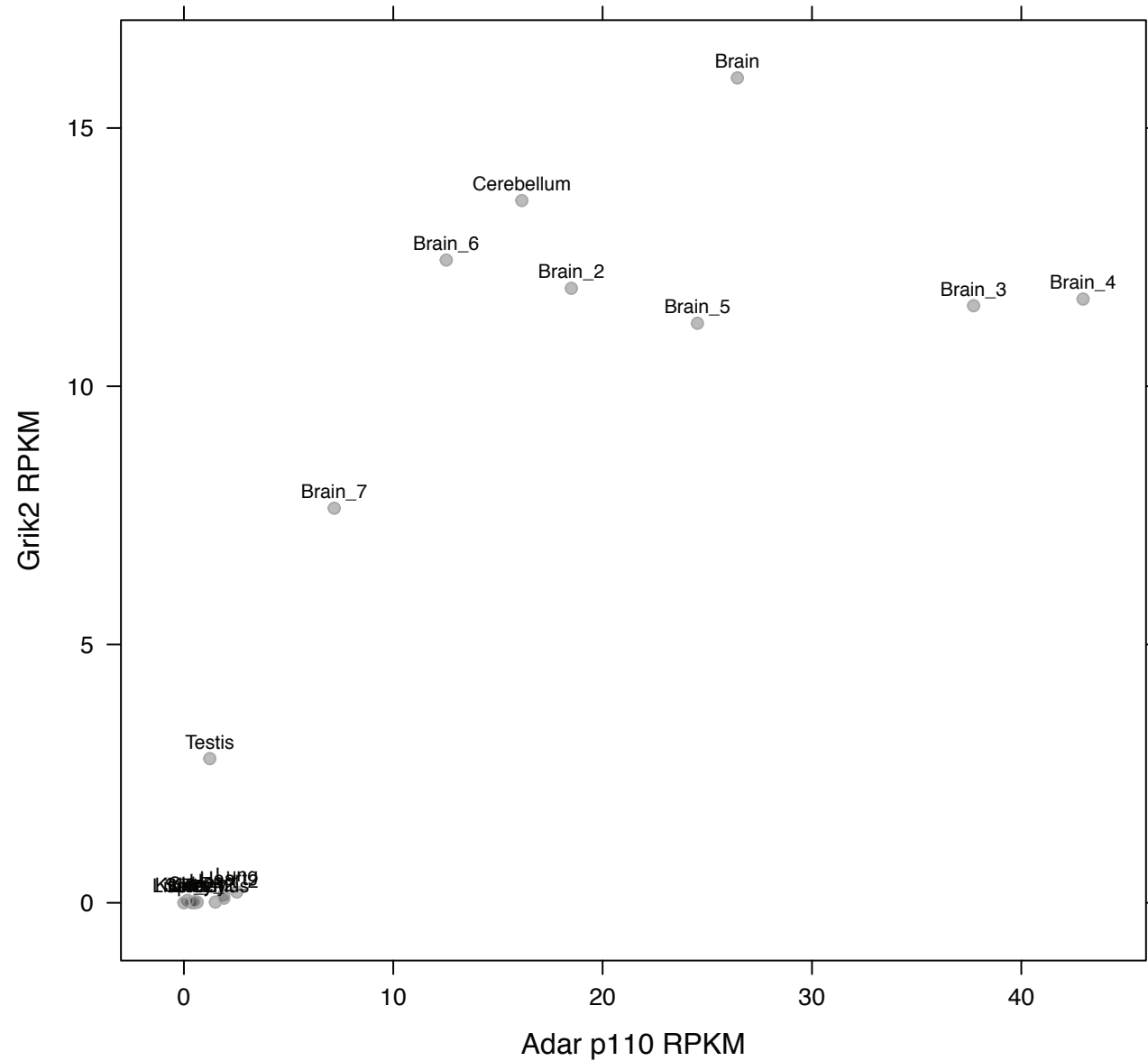**D**

**Rbbp4**  
 $\rho = 0.281$  pvalue = 0.243

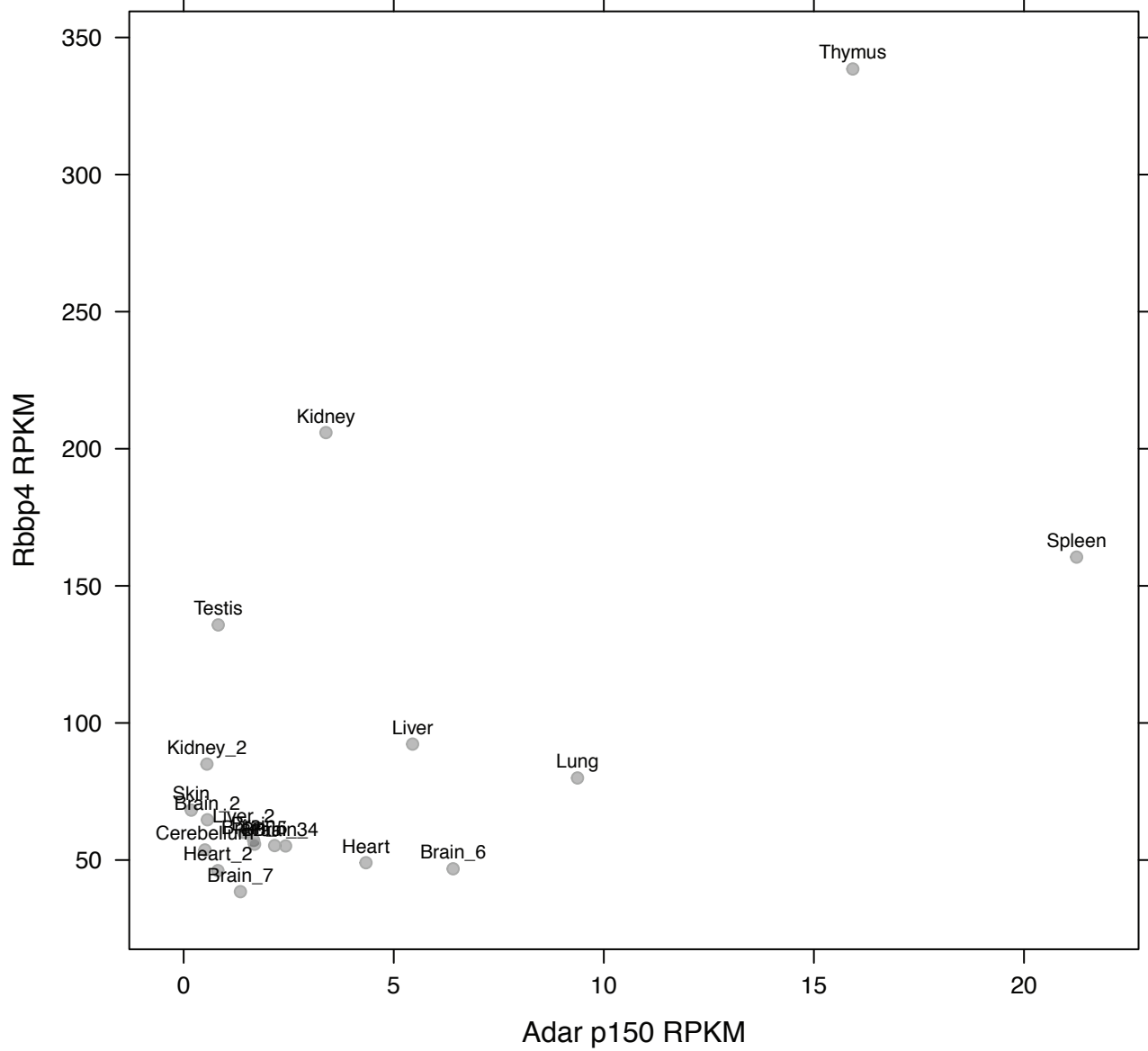

Supplement: Additional file 9 — Figure S9. After filtering, the breakdown of observed RDDs by type and strand. (PDF 4 kb) [file 12864_2015_2291_MOESM9_ESM.pdf]
