# Supplementary material for: Complex regulation of ADAR-mediated RNA-editing across tissues
Source: BMC Genomics. 2016 Jan 15;17:61. doi: 10.1186/s12864-015-2291-9 (PMC4714477; doi:10.1186/s12864-015-2291-9)
Supplement: Additional file 3 — Table S1. Gene Ontology analysis of non-synonymous A-to-I editing. (PDF 12 kb) [file 12864_2015_2291_MOESM3_ESM.pdf]

Table S1 Gene Ontology analysis of non-synonymous A-to-I editing

Tissue	GO ID	Term	p-value	Odds Ratio	Number of edited genes	Size of category
Brain	GO:0035235	ionotropic glutamate receptor signaling pathway	1.96E-08	227	4	16
	GO:0007270	neuron-neuron synaptic transmission	9.28E-06	41	4	70
	GO:0051966	regulation of synaptic transmission, glutamatergic	1.72E-05	80.8	3	26
	GO:0050806	positive regulation of synaptic transmission	8.00E-05	46.3	3	43
	GO:0031646	positive regulation of neurological system process	1.19E-04	40.2	3	49
	GO:0016050	vesicle organization	1.68E-04	35.6	3	55
	GO:0051969	regulation of transmission of nerve impulse	1.96E-04	18.1	4	152
	GO:0043113	receptor clustering	8.76E-04	56.8	2	22
	GO:0030521	androgen receptor signaling pathway	9.58E-04	54.1	2	23
	GO:0043525	positive regulation of neuron apoptotic process	1.04E-03	51.6	2	24
	GO:0044057	regulation of system process	1.10E-03	11.2	4	239
	GO:0016079	synaptic vesicle exocytosis	1.53E-03	42	2	29
	GO:0060079	regulation of excitatory postsynaptic membrane potential	1.42E-03	43.6	2	28
	GO:0017157	regulation of exocytosis	5.61E-03	20.9	2	56
	GO:0006898	receptor-mediated endocytosis	7.06E-03	18.5	2	63
	GO:0030522	intracellular receptor signaling pathway	7.50E-03	17.9	2	65
Testis	GO:0071705	nitrogen compound transport	1.41E-03	17.9	3	160
	GO:0051168	nuclear export	3.37E-03	28.7	2	60
	GO:0050658	RNA transport	4.31E-03	25.2	2	68
	GO:0006403	RNA localization	4.95E-03	23.4	2	73

In brain, six genes (Gria2, Gria4, Grik2, Grik5, Fcho2, Cadps) recur in most of the categories listed. Four genes were observed only once (Ar, Ahr, Kdm3a, and Stat3). Likewise in testis, 2 genes (Nup153 and Ddx25) were found in all reported categories. The gene Plekha8 was only observed once.
