# Supplementary material for: Complex regulation of ADAR-mediated RNA-editing across tissues
Source: BMC Genomics. 2016 Jan 15;17:61. doi: 10.1186/s12864-015-2291-9 (PMC4714477; doi:10.1186/s12864-015-2291-9)
Supplement: Additional file 6 — Figure S6. Sequence motifs (produced by the motifStack R package) surrounding editing sites, separated by tissue and genic context. (PDF 2389 kb) [file 12864_2015_2291_MOESM6_ESM.pdf]

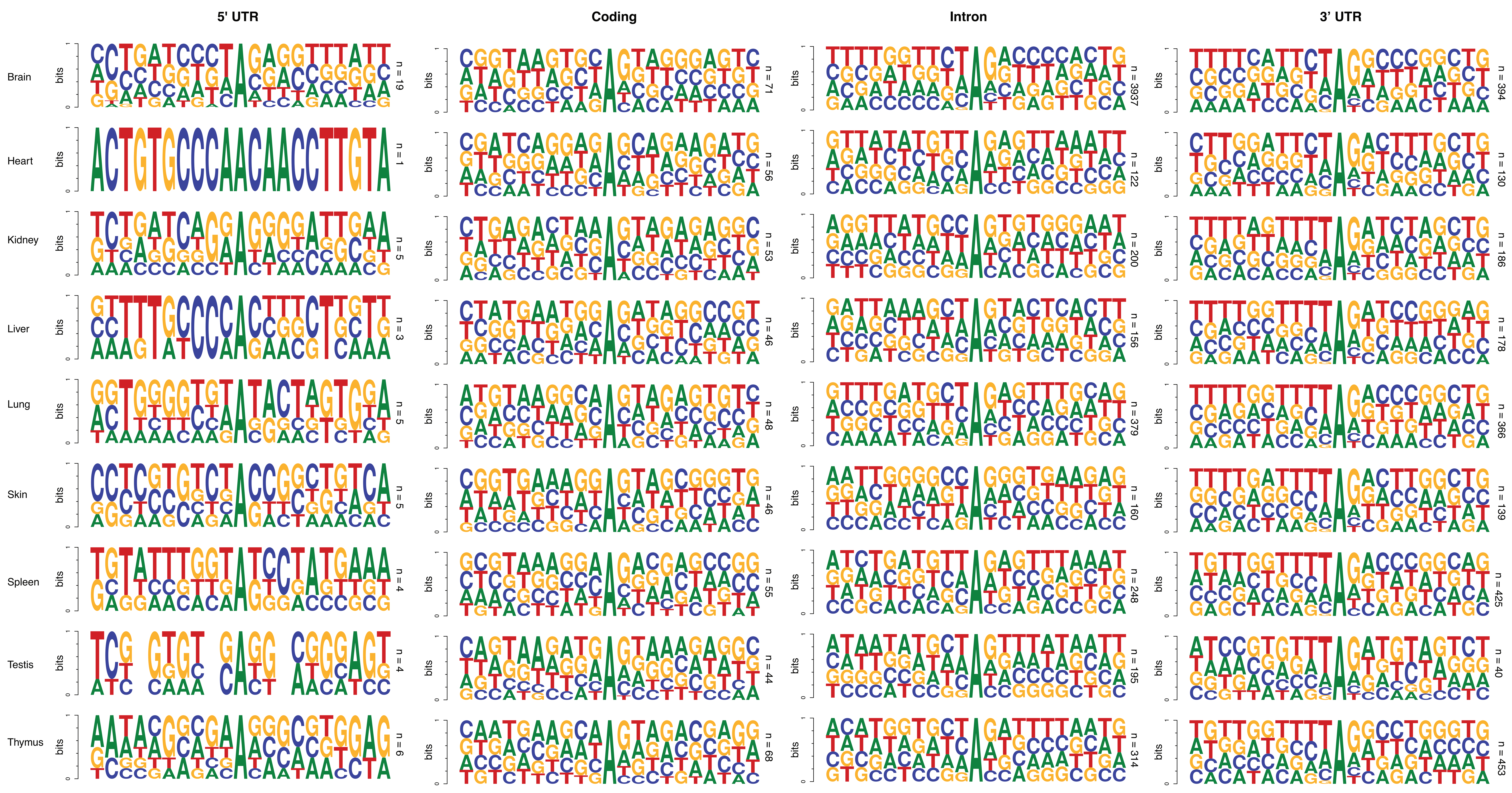
5' UTR
Coding
Intron
3' UTR
Brain
Heart
Kidney
Liver
Lung
Skin
Spleen
Testis
Thymus
bits
n = 19
n = 1
n = 5
n = 3
n = 5
n = 5
n = 4
n = 4
n = 6
n = 71
n = 56
n = 53
n = 46
n = 48
n = 46
n = 55
n = 44
n = 68
n = 3937
n = 122
n = 200
n = 156
n = 379
n = 160
n = 248
n = 195
n = 314
n = 394
n = 130
n = 186
n = 178
n = 366
n = 139
n = 425
n = 40
n = 453
